# Corrigendum

**DOI:** 10.1111/pbi.13185

**Published:** 2019-07-05

Tian, Y.‐S., Wang, B., Peng, R.‐H., Xu, J., Li, T., Fu, X.‐Y., Xiong, A.‐S., Gao, J.‐J., Yao, Q.‐H. Enhancing carotenoid biosynthesis in rice endosperm by metabolic engineering. *Plant Biotechnol. J*. **17**, 849‐851.

Figure 1d and 1f contains the exact same data. The corrected figure is shown below:

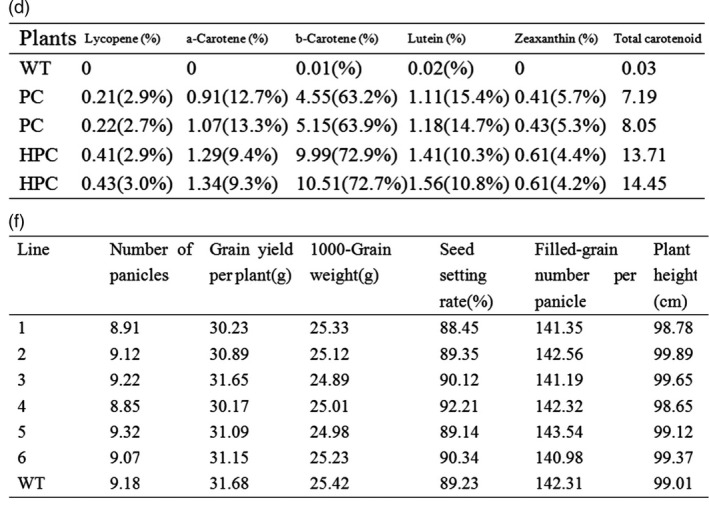

The authors apologize for this mistake.

